# Dislocation of the Head of the Femur, Separation of the Pubic Femoral Ligament from the Femur, and Rupture of the Capsular Ligament

**Published:** 1896-11

**Authors:** John J. Cattanach

**Affiliations:** New York City


### REPORTS OF CASES.

### A PECULIAR INJURY.

By JOHN J. CATTANACH, D.V.S.,
NEW YORK CITY.

I was called to attend a bay cob at 11 P.M., October 19, 1896, the property of a Mr. Day, at his stable, 31 East Forty-seventh Street, this city. The animal, showing symptoms of much pain, was standing in a single stall. On examination I found that he could not bear any weight on the near hind-leg, and that at times it appeared to be shorter than its fellow. The hock was turned inward and the toe outward, making the patella appear very prominent from a rear view. The coachman informed me that the horse was in the habit of being cast, and also a "chronic kicker." I at once came to the conclusion that the injury was in the hip, although the swelling appeared to be at the stifle. On revolving, extending the limb, etc., I could not discover any crepitation, and finally made a rectal examination without result. The animal was placed in slings the next morning, and in the afternoon showed a slightly diffused and painless swelling over the hip-joint. October 21st, a large and painful swelling appeared at the stifle, with much uneasiness, exhibited by the animal bearing heavily in the slings. October 22d, repeated rectal examination revealed nothing. The off-hind-leg showed great signs of weakness, and I advised the owner to have the horse destroyed, as I considered the case hopeless. My diagnosis was dislocation of the head of the femur, with fracture of the acetabulum.

Post-mortem showed complete dislocation of the head of the femur; complete separation of the pubic femoral ligament from the femur, and rupture of the capsular ligament. The acetabulum presented small pieces of bone chipped from the superior and external portion of the lip; the articular surface showing no sign of injury.

---

Racing a mile in 2.06¼, without harness, vehicle, or pacemaker, is the remarkable feat performed by the mare Marion Mills at Grand Rapids, Mich.

